# Physical punishment and child, adolescent, and adult outcomes in low- and middle-income countries: protocol for systematic review and meta-analysis

**DOI:** 10.1186/s13643-022-02154-5

**Published:** 2022-12-20

**Authors:** Jorge Cuartas, Elizabeth T. Gershoff, Drew Bailey, Dana C. McCoy

**Affiliations:** 1 Harvard Graduate School of Education, Cambridge, USA; 2 Centro de Estudios sobre Seguridad y Drogas (CESED), Universidad de los Andes, Carrera 1 # 18A – 12, Bogotá, Colombia; 3 Population Research Center, University of Texas at Austin, Austin, USA; 4 School of Education, University of California, Irvine, USA

**Keywords:** Violence against children, Physical punishment, Spanking, Meta-analysis, Systematic review, Low- and middle-income countries

## Abstract

**Background:**

Physical punishment at home and in schools is widespread around the world. Systematic reviews and meta-analyses have synthesized evidence, mostly from high-income countries (HICs), showing that physical punishment relates to multiple detrimental individual outcomes. Yet, less work has been done to synthesize the evidence on the association between physical punishment at home and schools and child, adolescent, and adult outcomes in low- and middle-income countries (LMICs), where more than 90% of children live and physical punishment is most socially normative and prevalent. In this manuscript, we present a protocol for a systematic review and meta-analysis on the characteristics of the research, associations, and variation in associations, between physical punishment at home and in schools and child, adolescent, and adult outcomes in LMICs.

**Methods:**

We will conduct a review of studies published in peer-reviewed journals using quantitative methods to assess the association between physical punishment in childhood and/or adolescence and individual outcomes in LMICs. We will search for studies in 10 different databases using keywords in English, Spanish, Portuguese, Arabic, and Chinese related to physical punishment. We will extract qualitative data from the studies and the statistics needed to transform all study-level effect sizes into standardized mean difference effect sizes. For the analyses, we will employ multi-level meta-analyses to use multiple effect sizes per study and leverage within-study variation as well as between study variation using moderation analysis. Besides the meta-analyses, we will also conduct a narrative synthesis of the findings.

**Discussion:**

The proposed systematic review and meta-analysis will provide timely evidence to inform global research, policy, and practice on the links between physical punishment and lifelong individual outcomes.

**Systematic review registration:**

PROSPERO CRD42022347346

**Supplementary Information:**

The online version contains supplementary material available at 10.1186/s13643-022-02154-5.

## Background

At least two out of three children younger than five living in low- and middle-income countries (LMICs) are exposed to physical punishment (also known as corporal punishment) in the home, early childhood care and education centers, or schools [1–3]. Extensive evidence from meta-analyses and reviews of cross-sectional and longitudinal studies—conducted mostly with samples from the USA and other high-income countries (HICs)—suggests that physical punishment relates to an array of detrimental child, adolescent, and adult outcomes [4–7]. In addition, the United Nations (UN) has firmly stated that physical punishment is a form of violence against children and a violation to children’s rights [8].

Despite evidence that physical punishment might be harmful, there is still some academic and extensive societal debate on the specific consequences of physical punishment. Most of the ongoing controversies are fueled by concerns of internal and external validity. First, there is disagreement on whether physical punishment causes worse child, adolescent, and adult outcomes versus whether confounding factors that plausibly influence both the likelihood of punishment and later individual outcomes (e.g., children’s initial levels of behavior) can fully explain observed associations between physical punishment and such outcomes [9–11]. Second, there has been a vast underrepresentation of samples from LMICs in prior meta-analyses and systematic reviews about physical punishment [5, 6, 9, 12]. This lack of studies in LMICs calls into question the generalizability of past findings given that 90% of children in the world live in LMICs and that physical punishment is prevalent in these countries [1]. Finally, most meta-analyses to date have focused on physical punishment at home and less is known about the potential links between physical punishment in schools and child, adolescent, and adult outcomes, with some recent exceptions [13, 14].

### Defining physical punishment considering a global perspective

Physical punishment is the use of physical force intended to cause some degree of pain or discomfort to correct or punish a child’s behavior [4, 15–17]. As such, physical punishment could vary in its frequency and severity, and could include actions like spanking, hitting a child with objects, or forcing a child to stay in an uncomfortable position, among others. A key feature of physical punishment is that adults have the intention to punish, correct, or control the child’s behavior [4, 16]. Some scholars have tried to distinguish between physical punishment (i.e., spanking) and abuse (e.g., hitting with hard objects or hitting the child frequently) based on how socially normative are different behaviors [18]. Yet, these US-centered distinctions, which have been common in the literature [9, 19], are far from universal and are difficult to operationalize (e.g., there is not a clear threshold that divides physical punishment from abuse and the same behaviors are not normative across countries/cultures). Despite this, to date little is known about the specific forms of physical punishment that have been studied in research conducted with samples from LMICs, where, in contrast with US-centered perspectives, behaviors like hitting with objects are as socially normative as spanking [3, 20].

### Theoretical and empirical links between physical punishment and child, adolescent, and adult outcomes

Developmental and educational theories and empirical evidence indicate that physical punishment can compromise children’s, adolescents’, and adults’ development, learning, and well-being through several biological and social mechanisms. According to traditional developmental and educational theories like social learning theory [21], social information processing theory [22], and attachment theory [23], by using physical punishment, caregivers and educators (intentionally or unintentionally) model aggression as a means to solve problems, inculcate in the child expectations of aggression, and erode the attachment bond, with downstream negative consequences on the relationship between children and their caregivers and children’s social-emotional development, behaviors, and mental health.

Contemporary neurodevelopmental models like the dimensional model of adversity [24] indicate that physical punishment can also affect children’s social-emotional and cognitive skills through neural mechanisms. The dimensional model of adversity predicts that exposure to threatening experiences, such as physical punishment, influence neural networks that facilitate the rapid identification of and response to environmental threats, including heightened response to negative emotional cues in brain regions that tend to underlie social and emotional processing and some cognitive functions [25, 26] Furthermore, the model predicts that the neural consequences of exposure to threatening experiences scale in relation to the severity of the threat involved. Finally, neurodevelopmental perspectives also indicates that threatening experiences might be more consequential if they occurred early in life, when the brain is more malleable and sensitive to experiences and contexts [27]. Consistent with these models, nascent evidence from neuroimaging studies shows associations between experiencing physical punishment early in life and atypical brain structure and function [28, 29], in ways that may lead to downstream behavioral, emotional, and cognitive consequences.

These theoretical perspectives also align with a growing number of studies from LMICs that have shown consistent associations between physical punishment and individual outcomes, which do not seem to vary across settings. A rapid review of 42 studies using samples from LMICs concluded that there is robust evidence on the associations between physical punishment and individual social-emotional and mental health outcomes, but the evidence for cognitive outcomes is scarcer and mixed [17]. In addition, studies using nationally representative samples for more than 49 countries across LMICs indicated that social normativeness does not modify the associations between physical punishment and individual outcomes [30, 31].

With these theoretical perspectives and empirical findings, we can hypothesize that: (1) physical punishment will likely be more strongly associated with social-emotional and mental health outcomes than with cognitive outcomes, (2) physical punishment could lead to stronger consequences if it occurs in early childhood relative to later in life (3) different forms of physical punishment could lead to different consequences that will likely scale in relation to the severity of threat involved, and (4) the same mechanisms linking physical punishment and individual outcomes might be relevant in different settings (e.g., in LMICs and HICs, between regions and countries, or if physical punishment takes place at home vs. schools).

### Issues of internal validity and effect sizes

Despite consistent evidence on the associations between physical punishment and negative individual outcomes, establishing credible causal links between physical punishment and child, adolescent, and adult outcomes is not straightforward. It would be unethical to randomly assign children to physical punishment vs. non-physical punishment conditions and to date it has proven impossible to identify arguable exogenous sources of variation for physical punishment to conduct instrumental variables or regression discontinuity designs. For example, programs aimed at preventing physical punishment tend to include other components that might promote positive parenting (for example, content on the importance of play and/or emotional communication), therefore making them endogenous (see as examples the ACT Raising Safe Kids program [32], the Irie Toolbox [33, 34], and Parenting for Lifelong Health [35, 36]). Therefore, researchers have mostly relied on observational designs, with some exceptions using fixed effects models [11, 37] and matching methods, in an attempt to improve the internal validity of estimates [38, 39]. Yet, none of these approaches rules out all potential confounders (i.e., characteristics that might simultaneously affect physical punishment and outcomes), even if researchers have longitudinal data [38, 40]. Failing to control for most confounders will likely lead to overestimating the association between physical punishment and different outcomes. For example, maternal depression will likely have a positive correlation with physical punishment and negative association with individual outcomes. As such, failing to account for it may lead to an artificially inflated estimate of the relation between physical punishment and individual outcomes. A similar situation will arise with variables related to socioeconomic status (SES), self-efficacy, and even genetics.

Given these challenges to establishing causal links between physical punishment (and other developmental characteristics/exposures) and child, adolescent, and adult outcomes, researchers have increasingly recognized the importance of strong theory and assessing the sensitivity or robustness of estimates to the inclusion of covariates, multiple methodological approaches, and different identifying assumptions [11, 38, 41]. While even meta-analyses of physical punishment have recognized the importance of sensitivity/robustness checks in research on the consequences of physical punishment, to date all meta-analyses have included only one effect size per outcome per study, therefore making it impossible to test how robust are effect sizes within studies.

Besides allowing to assess the robustness of estimates, the inclusion of more than one effect size per outcome per study is useful to exploit all available data, increasing statistical power and leveraging informative within-study variability. Including multiple effect sizes in a meta-analysis is not entirely straightforward, as conventional meta-analytic methods assume independence of effect sizes. However, multiple effect sizes from the same study (e.g., different outcomes) are likely to be non-independent for different reasons, including correlations between sampling errors (due to the use of same sample) or nesting within the primary study [42]. Under such circumstances, the results from conventional meta-analytic methods are inappropriate and could even be misleading [42, 43] and researchers have recommended the use of multilevel random-effects models to analyze datasets that include more than one effect size per study [43].

### The proposed systematic review and meta-analysis

The proposed study has two main objectives. The first aim is to conduct a systematic review of the literature examining the associations between physical punishment in childhood or adolescence and child, adolescent, and adult outcomes in LMICs to describe the quantity and characteristics of studies, including geographic distribution, definitions of physical punishment used, methodological approaches, and main findings, among other basic characteristics. The second objective is to conduct a series of meta-analyses of the associations between physical punishment and child, adolescent, and adult outcomes in LMICs. We will conduct searches in multiple languages and databases to find more studies from LMICs in addition to those considered in prior meta-analyses. Furthermore, we will include all relevant effect sizes and use state-of-the-art multilevel random effects models to analyze the data. In addition, these meta-analyses will, for the first time in the literature, include physical punishment both at home and in schools. Finally, we will conduct moderation analyses to assess variability in the links between physical punishment and child, adolescent, and adult outcomes in LMICs.

### Research questions and hypotheses

**RQ1:** What are the main characteristics of the published research on the associations between physical punishment and child, adolescent, and adult outcomes in LMICs regarding (a) geographic distribution, (b) the different forms of physical punishment that have been studied, and (c) methodological approaches?

We hypothesize that prior research (a) has not been widespread in different LMICs, but is scarce and has concentrated in specific countries, (b) has likely examined multiple forms of physical punishment, including spanking, hitting children with objects, and pinching the child, among others, and (c) has mostly relied on linear regression models with conventional covariate adjustment.

**RQ2:** What are the average associations between physical punishment and a range of child, adolescent, and adult outcomes in LMICs?

We hypothesize that all forms of physical punishment will associate with detrimental individual outcomes.

**RQ3.** Do the associations between physical punishment and child, adolescent, and adult outcomes vary by (a) different forms of physical punishment (e.g., spanking, hitting a child with objects), (b) developmental period at time of physical punishment [0–2 years; 3–5 years; 6–10 years; +10 years], (c) whether punishment occurred in homes or in schools, (d) region (i.e., East Asia and Pacific, Europe and Central Asia, Latin America & the Caribbean, Middle East and North Africa, North America, South Asia, Sub-Saharan Africa) or country income group (i.e., low-income, lower-middle income, upper-middle income), and (e) methodological approach (e.g., data structure and analytic strategy)?

We hypothesize that the association between physical punishment and individual outcomes will (a) vary according to different forms of physical punishment, (b) be stronger if physical punishment took place early in life relative to later developmental periods, (c) be similar regardless of whether punishment takes place in homes or in schools, (d) not vary across countries or regions, and (e) will be stronger in studies with weaker internal validity (e.g., cross-sectional, observational with poor covariates) relative to more internally valid studies (e.g., longitudinal, rich set of covariates, experimental, quasi-experimental) due to issues of selection bias.

## Methods

### Protocol registration and reporting

We followed the Preferred Reporting Items for Systematic Reviews and Meta-Analysis for Protocols (PRISMA-P) to develop this protocol (see Additional file 1: Appendix 1). This systematic review was registered in the International Prospective Register of Systematic Reviews (PROSPERO) on August 1, 2022.

### Inclusion and exclusion criteria

Table 1 presents the inclusion criteria for studies in the review, considering the PICO framework. The systematic review and meta-analysis will assess the average association (and variation in such associations by characteristics listed above) between physical punishment at home and schools and child, adolescent, and adult outcomes in LMICs.

**Table 1:** Inclusion criteria

	Included	Excluded
Aim	• Assess the association between any form of physical punishment and any child, adolescent, and/or adult outcome.	• Only assess the association between non-physical forms of discipline (e.g., psychological aggression, time-out) and any child outcome.
Study type	• Quantitative (experimental, quasi-experimental, observational) • Access to information to calculate effect sizes	• Qualitative, theoretical, case study • No access to information to calculate effect sizes
Publication date	• 2002–2022	• Published before 2002
Publication type	• Peer-reviewed journal	• Gray literature (e.g., dissertations, working papers)
Population	• Children, adolescents, and adults who experienced physical punishment in childhood or adolescence (<18 years of age) living in LMICs	• Children, adolescents, and adults living in high-income countries
Exposure	• Exposure to or frequency/severity of any form of physical punishment	• Physical violence that is not used for the purpose of controlling the child’s behavior • Beliefs or attitudes towards physical punishment (rather than exposure) • Indices that do not allow independent measurement of physical punishment and outcome (i.e., same data source)
Comparator	• Children, adolescent or adults who were not exposed to or were exposed less frequently or to less severe physical punishment	• No estimate for the association between physical punishment and outcome
Outcomes	• Any individual child, adolescent or adult outcome	• No outcomes
Language of publication	• Any	• None

### Study type

The review will consider studies published in peer-reviewed journals employing quantitative methods, including experimental, quasi-experimental (e.g., instrumental variables, difference-in-differences, matching), and observational approaches. Following prior meta-analyses on physical punishment and child, adolescent, and adult outcomes [5], we will exclude gray literature (e.g., dissertations, unpublished manuscripts) and qualitative, theoretical, and case studies. To be considered for inclusion, the studies should provide sufficient basic information to confidently calculate effect sizes (ES). If information to calculate ES is unavailable, we will contact the corresponding authors via email to request the information. If we do not receive a reply in 2 weeks to respond the original request, we will send a reminder and extend with another week. If we do not receive a reply, we will exclude the study.

### Population

The population will be restricted to children, adolescents, and adults living in LMICs. We will consider all countries that were categorized as LMICs in the period of the study (i.e., 2002 onwards) by the World Bank Country and Lending Groups [44].

### Exposure

This systematic review and meta-analysis will focus on all forms of physical punishment, following the definitions discussed above. In the review we will include studies that measure any form of physical punishment vs. no exposure to physical punishment and those that use continuous measures of physical punishment frequency or severity. Studies will be included as long as authors assert that they are measuring physical punishment. We will exclude studies that measure beliefs of or attitudes towards physical punishment rather than actual exposure to physical punishment, and studies that use only indices (e.g., Adverse Childhood Experiences - ACEs) and do not allow to measure physical punishment independently from other adversities or forms of violence.

### Comparator

The review will include studies that compare children exposed to physical punishment to a comparison group of children, adolescents or adults who were never exposed to physical punishment or were exposed less to less frequent or severe physical punishment.

### Outcomes

We will follow prior meta-analyses [4, 5, 9] of the association between physical punishment (or spanking, specifically) and child, adolescent, and adult outcomes to focus on outcomes related to (1) externalizing behavior problems (e.g., aggression), (2) internalizing behavior problems, (3) mental health problems, (4) alcohol or substance abuse, (5) parent-child relationships, (6) cognitive development (including academic achievement), (7) social-emotional development (e.g., self-esteem and self-regulation), (8) probability of being a victim of physical abuse, and (9) support for physical punishment and other forms of violence. We may add new or collapse some of the abovementioned outcome categories after conducting the data extraction.

### Search strategy

We consulted five librarians from Harvard University to identify relevant databases that might include studies from LMICs. Furthermore, we searched journals in Arabic, Chinese, Spanish, Portuguese, French, Hindi, and Swahili specialized in psychology, education, medicine, and public health in UlrichsWeb [45]. Subsequently, we identified the indexation of the journals and included additional databases as long as they had a Thesaurus (i.e., specialized subject terms) to ensure reproducibility of our searches. With such information, we decided to search the following 10 databases: (1) APA PsycInfo, (2) PubMed, (3) EMBASE, (4) ERIC, (5) Sociological Abstracts, (6) Global Health, (7) CINAHL Plus with full text, (8) Academic Search Premier, (9) Bibliography of Asian Studies, and (10) Education Source.

We will search the databases for the following keywords in English, Spanish, Portuguese, French, Arabic, and Chinese in titles and abstracts: spank*, corporal punishment*, physical punishment*, physical disciplin*, corporal disciplin*, harsh punishment*, harsh disciplin*, and smack* (see Additional file 2: Appendix 2 for the search code). In all searches, we will use the filters for publication date (i.e., published after 2002) and type of publication (i.e., peer-reviewed journals).

In addition, we will consider all the studies that met our inclusion criteria from prior published systematic reviews and meta-analyses on the association between physical punishment and child, adolescent, and adult outcomes [4–6, 9, 12–14, 46, 47].

### Screening and full text-review

We will export all prospective studies to Covidence (www.covidence.org) in order to ensure reproducibility of the decision process. Before title and abstract screening, we will remove all duplicates from the Covidence library. Two reviewers (JC and a trained research assistant) will independently examine all titles and abstracts following pre-established inclusion criteria. All disagreements will be resolved by consensus through discussion among the reviewers and a third reviewer (ETG) will help resolve remaining conflicts if consensus is not reached. Subsequently, JC and a trained research assistant will independently review the full text of the remaining records considering the inclusion criteria, and all disagreements will be solved following the same procedures of the title and abstract screening phase.

### Data extraction

At least two reviewers will extract data for the studies meeting the inclusion criteria using a pre-piloted standardized data extraction template. We will extract qualitative data from the studies in Covidence and the statistics needed to calculate ES in an Excel spreadsheet. Among other information, we will extract data on:

1. Basic details about the study (e.g., authors, year of publication)
2. Details related to sample characteristics (e.g., country, sample size, distribution by sex, age)
3. Data structure (e.g., cross-sectional, longitudinal, experimental, retrospective)
4. Methodological approach (e.g., experimental or observational)
5. Covariates included in the models, if any
6. Measure of physical punishment (e.g., observation, parent report, child report, child report retrospective, both parent and child report)
7. Definition of physical punishment used in the study
8. Setting where physical punishment occurred (i.e., home or school)
9. If available, prevalence of physical punishment in the sample
10. Characteristics of the measure of physical punishment (e.g., frequency, severity, period in which spanking was administered [last week, ever in life, last month, last year], developmental period at time of physical punishment [0–2 years; 3–5 years; 6–10 years; +10 years])
11. Measure of outcome(s) (e.g., direct assessment, parent report, child report)
12. Definition of outcome and age at which outcome was measured
13. Independence of measures of physical punishment and outcome (e.g., same or different rater)
14. Narrative synthesis of main results and moderation (e.g., variation in the association between physical punishment and outcomes due to sex, caregiver’s education or household wealth, among other characteristics)
15. Statistics to calculate effect sizes (e.g., means, standard deviations) for the association between physical punishment and outcomes

### Effect size calculation

At least two reviewers will extract all the available relevant ES for each study. We will include all ES that allow us to compare between (1) unadjusted and adjusted models, (2) models with different identifying assumptions, and (3) associations between physical punishment and child, adolescent, and adult outcomes in different countries. We will transform all study-level effect sizes into standardized mean different effect sizes (i.e., Cohen’s *d*). For studies that report effect sizes as group comparisons (e.g., exposed to physical punishment vs. not exposed) we will use Cohen’s formula $d=\frac{\mu_1-{\mu}_2}{\sigma }$, where *μ*_1_ is the mean for the group exposed to physical punishment, *μ*_2_ the mean of the comparison group, and *σ* the pooled standard deviation. For studies that do not report effect sizes as group comparisons (e.g., frequency or severity of physical punishment), we will follow the procedures presented in Borenstein and colleagues [48] to convert correlations and other quantitative measures of associations to Cohen’s *d* effect sizes.

### Data synthesis

We will begin by conducting a narrative synthesis of the findings, including (1) definitions of physical punishment used in research from LMICs, (2) geographic distribution and methodological approaches, (3) main findings, and (4) results of moderation analyses.

It is likely that many studies included in the review will include more than one effect size (e.g., multiple outcomes, unadjusted and adjusted, for two models with different identifying assumptions). The sampling errors of multiple effect sizes from a single study might be correlated due to nesting within the same study and usage of the same sample, thus violating the assumption of independence and threatening the validity of the meta-analyses [42]. One solution proposed in prior meta-analysis on physical punishment and individual outcomes was to select one effect size per sample or averaging across effect sizes [9, 46]. Yet, such practice leads to loss of information that decreases statistical power and might obscure important variability to assess the sensitivity or robustness of the estimated effect sizes.

We will follow current best-practice recommendations [42, 43] to dealing with multiple effect sizes and non-independence and will employ multilevel random effects models to analyze the data. Multilevel random effects models will allow us to include all relevant effect sizes and model the dependence between effects within the same sample or study [43]. We will employ random effects, rather than fixed effects models, under the assumption that differences between studies in observed effect sizes might be due to both “real” differences and measurement error (rather than exclusively measurement error) [48]. The random effects meta-analysis will allow us to estimate a mean effect size along with a 95% CI around such estimate and a measure of heterogeneity (*I*^2^) to assess the extent to which effect sizes differ between studies.

The moderation analysis will depend mostly on the final sample of studies and the extent to which the sample of studies provides enough statistical power to do such analyses. Given that there are no prior systematic reviews about physical punishment and child, adolescent, and adult outcomes in LMICs, we do not have an a priori estimate of the number of studies we will likely identify. In any case, we expect to do some subgroup analysis according to characteristics listed in the third research question listed above. We will assess risk of bias and quality of the evidence through moderation analysis in order to assess whether the links between physical punishment and individual outcomes is robust to different methodological approaches.

### Dissemination plans

We will submit the findings of the review for publication to a peer-reviewed journal. We expect to disseminate findings from the review in blogs, conferences, and other outlets.

## Discussion

Physical punishment constitutes a violation to children’s rights [15] and a setback to global policy goals, as one indicator for the UN Sustainable Development Goals (SDGs) is the proportion of children exposed to physical punishment [49]. Furthermore, extensive evidence, mostly from the USA and other HICs [5, 6], shows consistent links between physical punishment at home and detrimental outcomes throughout the lifespan. Nonetheless, less work has been done to synthesize the evidence on the association between physical punishment at homes and schools and child, adolescent, and adult outcomes in LMICs, where more than 90% of children live [50] and physical punishment is most socially normative and prevalent [1]. In addition, there is a need for new analyses that employ state-of-the-art meta-analytic methods to strengthen the claim that physical punishment might be detrimental for individuals, assessing robustness of estimates and variation between studies and groups.

The proposed meta-analysis seeks to contribute to filling the above-mentioned gaps by focusing on studies conducted with samples from LMICs, considering physical punishment that takes place at home and schools, considering all relevant effect sizes from each study, and using novel multilevel random effects modeling. Using such information, the meta-analysis will not only estimate the average association between physical punishment and different outcomes but also assess within and between study variability in effect sizes, as well as moderation due to study, individual, and contextual characteristics.

While the meta-analysis will have several strengths, we also anticipate some limitations. First, we do not expect finding any experimental studies conducted with samples from LMICs, and we expect few studies with longitudinal or quasi-experimental designs. As such, we anticipate that most studies will be cross-sectional, observational, and employ conventional covariate adjustment. Yet, we will assess within-study variability and assess moderation due to data structure and methodological approach to assess the sensitivity of estimates. Second, we anticipate that several included studies will have measurement issues, including parent reported measures of physical punishment and measures that are limited regarding temporality (e.g., physical punishment in the past week or month). However, measurement error due to these issues might drive the estimates against our stated hypotheses (i.e., will lead to coefficient attenuation, or an underestimation of the “true” association between physical punishment and individual outcomes). Finally, given the vast underrepresentation of samples from LMICs in research about physical punishment, we anticipate some inconveniences with availability of studies/information and statistical power to conduct some moderation analyses.

To conclude, this meta-analysis will offer new evidence on the potential consequences of physical punishment in LMICs. The meta-analysis will help identify some strengths and weaknesses of existing evidence in order to inform future research on the links between physical punishment and child, adolescent, and adult outcomes. Furthermore, the new evidence will inform policies aimed at protecting children from psychosocial threats to their development, as well as practice regarding caregiving in LMICs.

## Supplementary Information

**Additional file 1:** PRISMA-P.

**Additional file 2:** CODES.
