# Supplementary material for: Physical punishment and child, adolescent, and adult outcomes in low- and middle-income countries: protocol for systematic review and meta-analysis
Source: Syst Rev. 2022 Dec 20;11:276. doi: 10.1186/s13643-022-02154-5 (PMC9769004; doi:10.1186/s13643-022-02154-5)
Supplement: Supplementary file 2 — Additional file 2: CODES. [file 13643_2022_2154_MOESM2_ESM.docx]

**Appendix B. code**

**PsycInfo**

TI ( "spank*" OR "corporal punishment*" OR "physical punishment*" OR "physical disciplin*" OR "corporal disciplin*" OR "harsh punishment*" OR "harsh disciplin*" OR “smack*” OR “punition physique” OR “punition corporelle” OR “châtiment corporel” OR “fessée” OR “castigo fisico” OR “castigos fisicos” OR “castigo corporal” OR “castigos corporales” OR “palmada*” OR “disciplina fisica” OR “disciplina violenta” OR "Surra*" OR "punição corporal*" OR "punição física*" OR "agressão corporal*" OR "espancar*" OR "Surra corretiva*" OR "castigo físico*" OR "palmadas pedagógicas" OR "castigo corporal*" OR “打骂” OR “打屁股” OR “体罚” OR “العقاب البدني” OR “العقوبة البدنية” OR “العقاب الجسدي” OR “التأديب الجسدي” OR “التأديب بالعنف” OR “العقوبة بالعنف” ) OR AB ( "spank*" OR "corporal punishment*" OR "physical punishment*" OR "physical disciplin*" OR "corporal disciplin*" OR "harsh punishment*" OR "harsh disciplin*" OR “smack*” OR “punition physique” OR “punition corporelle” OR “châtiment corporel” OR “fessée” OR “castigo fisico” OR “castigos fisicos” OR “castigo corporal” OR “castigos corporales” OR “palmada*” OR “disciplina fisica” OR “disciplina violenta” OR "Surra*" OR "punição corporal*" OR "punição física*" OR "agressão corporal*" OR "espancar*" OR "Surra corretiva*" OR "castigo físico*" OR "palmadas pedagógicas" OR "castigo corporal*" OR “打骂” OR “打屁股” OR “体罚” OR “العقاب البدني” OR “العقوبة البدنية” OR “العقاب الجسدي” OR “التأديب الجسدي” OR “التأديب بالعنف” OR “العقوبة بالعنف” )

**PubMed (MEDLINE)**

"spank*"[Title/Abstract] OR "corporal punishment*"[Title/Abstract] OR "physical punishment*"[Title/Abstract] OR "physical disciplin*"[Title/Abstract] OR "corporal disciplin*"[Title/Abstract] OR "harsh punishment*"[Title/Abstract] OR "harsh disciplin*"[Title/Abstract] OR "smack*" OR "punition physique" OR "punition corporelle" OR "châtiment corporel" OR "fessée" OR "castigo fisico" OR "castigos fisicos" OR "castigo corporal" OR "castigos corporales" OR "palmada*" OR "disciplina fisica" OR "disciplina violenta" OR "Surra*"[Title/Abstract] OR "punição corporal*"[Title/Abstract] OR "punição física*"[Title/Abstract] OR "agressão corporal*"[Title/Abstract] OR "espancar*"[Title/Abstract] OR "Surra corretiva*"[Title/Abstract] OR "castigo físico*"[Title/Abstract] OR "palmadas pedagógicas"[Title/Abstract] OR "castigo corporal*"[Title/Abstract] OR "打骂" OR "打屁股" OR "体罚" OR "العقاب البدني" OR "العقوبة البدنية" OR "العقاب الجسدي" OR "التأديب الجسدي" OR "التأديب بالعنف" OR "العقوبة بالعنف"

**EMBASE**

'spank*':ab,ti OR 'corporal punishment*':ab,ti OR 'physical punishment*':ab,ti OR 'physical disciplin*':ab,ti OR 'corporal disciplin*':ab,ti OR 'harsh punishment*':ab,ti OR 'harsh disciplin*':ab,ti OR 'smack*':ab,ti OR 'punition physique':ab,ti OR 'punition corporelle':ab,ti OR 'châtiment corporel':ab,ti OR 'fessée':ab,ti OR 'castigo fisico':ab,ti OR 'castigos fisicos':ab,ti OR 'castigo corporal':ab,ti OR 'castigos corporales':ab,ti OR 'palmada*':ab,ti OR 'disciplina fisica':ab,ti OR 'disciplina violenta':ab,ti OR 'surra*':ab,ti OR 'punição corporal*':ab,ti OR 'punição física*':ab,ti OR 'agressão corporal*':ab,ti OR 'espancar*':ab,ti OR 'surra corretiva*':ab,ti OR 'castigo físico*':ab,ti OR 'palmadas pedagógicas':ab,ti OR 'castigo corporal*':ab,ti OR '打骂':ab,ti OR '打屁股':ab,ti OR '体罚':ab,ti OR 'العقاب البدني':ab,ti OR 'العقوبة البدنية':ab,ti OR 'العقاب الجسدي':ab,ti OR 'التأديب الجسدي':ab,ti OR 'التأديب بالعنف':ab,ti OR 'العقوبة بالعنف':ab,ti

**ERIC**

TI ( "spank*" OR "corporal punishment*" OR "physical punishment*" OR "physical disciplin*" OR "corporal disciplin*" OR "harsh punishment*" OR "harsh disciplin*" OR “smack*” OR “punition physique” OR “punition corporelle” OR “châtiment corporel” OR “fessée” OR “castigo fisico” OR “castigos fisicos” OR “castigo corporal” OR “castigos corporales” OR “palmada*” OR “disciplina fisica” OR “disciplina violenta” OR "Surra*" OR "punição corporal*" OR "punição física*" OR "agressão corporal*" OR "espancar*" OR "Surra corretiva*" OR "castigo físico*" OR "palmadas pedagógicas" OR "castigo corporal*" OR “打骂” OR “打屁股” OR “体罚” OR “العقاب البدني” OR “العقوبة البدنية” OR “العقاب الجسدي” OR “التأديب الجسدي” OR “التأديب بالعنف” OR “العقوبة بالعنف” ) OR AB ( "spank*" OR "corporal punishment*" OR "physical punishment*" OR "physical disciplin*" OR "corporal disciplin*" OR "harsh punishment*" OR "harsh disciplin*" OR “smack*” OR “punition physique” OR “punition corporelle” OR “châtiment corporel” OR “fessée” OR “castigo fisico” OR “castigos fisicos” OR “castigo corporal” OR “castigos corporales” OR “palmada*” OR “disciplina fisica” OR “disciplina violenta” OR "Surra*" OR "punição corporal*" OR "punição física*" OR "agressão corporal*" OR "espancar*" OR "Surra corretiva*" OR "castigo físico*" OR "palmadas pedagógicas" OR "castigo corporal*" OR “打骂” OR “打屁股” OR “体罚” OR “العقاب البدني” OR “العقوبة البدنية” OR “العقاب الجسدي” OR “التأديب الجسدي” OR “التأديب بالعنف” OR “العقوبة بالعنف” )

**Sociological Abstracts**

ab("spank*" OR "corporal punishment*" OR "physical punishment*" OR "physical disciplin*" OR "corporal disciplin*" OR "harsh punishment*" OR "harsh disciplin*" OR “smack*” OR “punition physique” OR “punition corporelle” OR “châtiment corporel” OR “fessée” OR “castigo fisico” OR “castigos fisicos” OR “castigo corporal” OR “castigos corporales” OR “palmada*” OR “disciplina fisica” OR “disciplina violenta” OR "Surra*" OR "punição corporal*" OR "punição física*" OR "agressão corporal*" OR "espancar*" OR "Surra corretiva*" OR "castigo físico*" OR "palmadas pedagógicas" OR "castigo corporal*" OR “打骂” OR “打屁股” OR “体罚” OR “العقاب البدني” OR “العقوبة البدنية” OR “العقاب الجسدي” OR “التأديب الجسدي” OR “التأديب بالعنف” OR “العقوبة بالعنف”) OR ti("spank*" OR "corporal punishment*" OR "physical punishment*" OR "physical disciplin*" OR "corporal disciplin*" OR "harsh punishment*" OR "harsh disciplin*" OR “smack*” OR “punition physique” OR “punition corporelle” OR “châtiment corporel” OR “fessée” OR “castigo fisico” OR “castigos fisicos” OR “castigo corporal” OR “castigos corporales” OR “palmada*” OR “disciplina fisica” OR “disciplina violenta” OR "Surra*" OR "punição corporal*" OR "punição física*" OR "agressão corporal*" OR "espancar*" OR "Surra corretiva*" OR "castigo físico*" OR "palmadas pedagógicas" OR "castigo corporal*" OR “打骂” OR “打屁股” OR “体罚” OR “العقاب البدني” OR “العقوبة البدنية” OR “العقاب الجسدي” OR “التأديب الجسدي” OR “التأديب بالعنف” OR “العقوبة بالعنف”)

**Global Health**

TI ( "spank*" OR "corporal punishment*" OR "physical punishment*" OR "physical disciplin*" OR "corporal disciplin*" OR "harsh punishment*" OR "harsh disciplin*" OR “smack*” OR “punition physique” OR “punition corporelle” OR “châtiment corporel” OR “fessée” OR “castigo fisico” OR “castigos fisicos” OR “castigo corporal” OR “castigos corporales” OR “palmada*” OR “disciplina fisica” OR “disciplina violenta” OR "Surra*" OR "punição corporal*" OR "punição física*" OR "agressão corporal*" OR "espancar*" OR "Surra corretiva*" OR "castigo físico*" OR "palmadas pedagógicas" OR "castigo corporal*" OR “打骂” OR “打屁股” OR “体罚” OR “العقاب البدني” OR “العقوبة البدنية” OR “العقاب الجسدي” OR “التأديب الجسدي” OR “التأديب بالعنف” OR “العقوبة بالعنف” ) OR AB ( "spank*" OR "corporal punishment*" OR "physical punishment*" OR "physical disciplin*" OR "corporal disciplin*" OR "harsh punishment*" OR "harsh disciplin*" OR “smack*” OR “punition physique” OR “punition corporelle” OR “châtiment corporel” OR “fessée” OR “castigo fisico” OR “castigos fisicos” OR “castigo corporal” OR “castigos corporales” OR “palmada*” OR “disciplina fisica” OR “disciplina violenta” OR "Surra*" OR "punição corporal*" OR "punição física*" OR "agressão corporal*" OR "espancar*" OR "Surra corretiva*" OR "castigo físico*" OR "palmadas pedagógicas" OR "castigo corporal*" OR “打骂” OR “打屁股” OR “体罚” OR “العقاب البدني” OR “العقوبة البدنية” OR “العقاب الجسدي” OR “التأديب الجسدي” OR “التأديب بالعنف” OR “العقوبة بالعنف” )

**CINAHL Plus with Full Text**

TI ( "spank*" OR "corporal punishment*" OR "physical punishment*" OR "physical disciplin*" OR "corporal disciplin*" OR "harsh punishment*" OR "harsh disciplin*" OR “smack*” OR “punition physique” OR “punition corporelle” OR “châtiment corporel” OR “fessée” OR “castigo fisico” OR “castigos fisicos” OR “castigo corporal” OR “castigos corporales” OR “palmada*” OR “disciplina fisica” OR “disciplina violenta” OR "Surra*" OR "punição corporal*" OR "punição física*" OR "agressão corporal*" OR "espancar*" OR "Surra corretiva*" OR "castigo físico*" OR "palmadas pedagógicas" OR "castigo corporal*" OR “打骂” OR “打屁股” OR “体罚” OR “العقاب البدني” OR “العقوبة البدنية” OR “العقاب الجسدي” OR “التأديب الجسدي” OR “التأديب بالعنف” OR “العقوبة بالعنف” ) OR AB ( "spank*" OR "corporal punishment*" OR "physical punishment*" OR "physical disciplin*" OR "corporal disciplin*" OR "harsh punishment*" OR "harsh disciplin*" OR “smack*” OR “punition physique” OR “punition corporelle” OR “châtiment corporel” OR “fessée” OR “castigo fisico” OR “castigos fisicos” OR “castigo corporal” OR “castigos corporales” OR “palmada*” OR “disciplina fisica” OR “disciplina violenta” OR "Surra*" OR "punição corporal*" OR "punição física*" OR "agressão corporal*" OR "espancar*" OR "Surra corretiva*" OR "castigo físico*" OR "palmadas pedagógicas" OR "castigo corporal*" OR “打骂” OR “打屁股” OR “体罚” OR “العقاب البدني” OR “العقوبة البدنية” OR “العقاب الجسدي” OR “التأديب الجسدي” OR “التأديب بالعنف” OR “العقوبة بالعنف” )

**Academic Search Premier**

TI ( "spank*" OR "corporal punishment*" OR "physical punishment*" OR "physical disciplin*" OR "corporal disciplin*" OR "harsh punishment*" OR "harsh disciplin*" OR “smack*” OR “punition physique” OR “punition corporelle” OR “châtiment corporel” OR “fessée” OR “castigo fisico” OR “castigos fisicos” OR “castigo corporal” OR “castigos corporales” OR “palmada*” OR “disciplina fisica” OR “disciplina violenta” OR "Surra*" OR "punição corporal*" OR "punição física*" OR "agressão corporal*" OR "espancar*" OR "Surra corretiva*" OR "castigo físico*" OR "palmadas pedagógicas" OR "castigo corporal*" OR “打骂” OR “打屁股” OR “体罚” OR “العقاب البدني” OR “العقوبة البدنية” OR “العقاب الجسدي” OR “التأديب الجسدي” OR “التأديب بالعنف” OR “العقوبة بالعنف” ) OR AB ( "spank*" OR "corporal punishment*" OR "physical punishment*" OR "physical disciplin*" OR "corporal disciplin*" OR "harsh punishment*" OR "harsh disciplin*" OR “smack*” OR “punition physique” OR “punition corporelle” OR “châtiment corporel” OR “fessée” OR “castigo fisico” OR “castigos fisicos” OR “castigo corporal” OR “castigos corporales” OR “palmada*” OR “disciplina fisica” OR “disciplina violenta” OR "Surra*" OR "punição corporal*" OR "punição física*" OR "agressão corporal*" OR "espancar*" OR "Surra corretiva*" OR "castigo físico*" OR "palmadas pedagógicas" OR "castigo corporal*" OR “打骂” OR “打屁股” OR “体罚” OR “العقاب البدني” OR “العقوبة البدنية” OR “العقاب الجسدي” OR “التأديب الجسدي” OR “التأديب بالعنف” OR “العقوبة بالعنف” )

**Bibliography of Asian Studies**  (can’t search abstracts here)

TI "spank*" OR "corporal punishment*" OR "physical punishment*" OR "physical disciplin*" OR "corporal disciplin*" OR "harsh punishment*" OR "harsh disciplin*" OR “smack*” OR “punition physique” OR “punition corporelle” OR “châtiment corporel” OR “fessée” OR “castigo fisico” OR “castigos fisicos” OR “castigo corporal” OR “castigos corporales” OR “palmada*” OR “disciplina fisica” OR “disciplina violenta” OR "Surra*" OR "punição corporal*" OR "punição física*" OR "agressão corporal*" OR "espancar*" OR "Surra corretiva*" OR "castigo físico*" OR "palmadas pedagógicas" OR "castigo corporal*" OR “打骂” OR “打屁股” OR “体罚” OR “العقاب البدني” OR “العقوبة البدنية” OR “العقاب الجسدي” OR “التأديب الجسدي” OR “التأديب بالعنف” OR “العقوبة بالعنف”

**Education Source**

AB ( "spank*" OR "corporal punishment*" OR "physical punishment*" OR "physical disciplin*" OR "corporal disciplin*" OR "harsh punishment*" OR "harsh disciplin*" OR “smack*” OR “punition physique” OR “punition corporelle” OR “châtiment corporel” OR “fessée” OR “castigo fisico” OR “castigos fisicos” OR “castigo corporal” OR “castigos corporales” OR “palmada*” OR “disciplina fisica” OR “disciplina violenta” OR "Surra*" OR "punição corporal*" OR "punição física*" OR "agressão corporal*" OR "espancar*" OR "Surra corretiva*" OR "castigo físico*" OR "palmadas pedagógicas" OR "castigo corporal*" OR “打骂” OR “打屁股” OR “体罚” OR “العقاب البدني” OR “العقوبة البدنية” OR “العقاب الجسدي” OR “التأديب الجسدي” OR “التأديب بالعنف” OR “العقوبة بالعنف” ) OR TI ( "spank*" OR "corporal punishment*" OR "physical punishment*" OR "physical disciplin*" OR "corporal disciplin*" OR "harsh punishment*" OR "harsh disciplin*" OR “smack*” OR “punition physique” OR “punition corporelle” OR “châtiment corporel” OR “fessée” OR “castigo fisico” OR “castigos fisicos” OR “castigo corporal” OR “castigos corporales” OR “palmada*” OR “disciplina fisica” OR “disciplina violenta” OR "Surra*" OR "punição corporal*" OR "punição física*" OR "agressão corporal*" OR "espancar*" OR "Surra corretiva*" OR "castigo físico*" OR "palmadas pedagógicas" OR "castigo corporal*" OR “打骂” OR “打屁股” OR “体罚” OR “العقاب البدني” OR “العقوبة البدنية” OR “العقاب الجسدي” OR “التأديب الجسدي” OR “التأديب بالعنف” OR “العقوبة بالعنف” )

**EconLit**

ab("spank*" OR "corporal punishment*" OR "physical punishment*" OR "physical disciplin*" OR "corporal disciplin*" OR "harsh punishment*" OR "harsh disciplin*" OR “smack*” OR “punition physique” OR “punition corporelle” OR “châtiment corporel” OR “fessée” OR “castigo fisico” OR “castigos fisicos” OR “castigo corporal” OR “castigos corporales” OR “palmada*” OR “disciplina fisica” OR “disciplina violenta” OR "Surra*" OR "punição corporal*" OR "punição física*" OR "agressão corporal*" OR "espancar*" OR "Surra corretiva*" OR "castigo físico*" OR "palmadas pedagógicas" OR "castigo corporal*" OR “打骂” OR “打屁股” OR “体罚” OR “العقاب البدني” OR “العقوبة البدنية” OR “العقاب الجسدي” OR “التأديب الجسدي” OR “التأديب بالعنف” OR “العقوبة بالعنف”) OR ti("spank*" OR "corporal punishment*" OR "physical punishment*" OR "physical disciplin*" OR "corporal disciplin*" OR "harsh punishment*" OR "harsh disciplin*" OR “smack*” OR “punition physique” OR “punition corporelle” OR “châtiment corporel” OR “fessée” OR “castigo fisico” OR “castigos fisicos” OR “castigo corporal” OR “castigos corporales” OR “palmada*” OR “disciplina fisica” OR “disciplina violenta” OR "Surra*" OR "punição corporal*" OR "punição física*" OR "agressão corporal*" OR "espancar*" OR "Surra corretiva*" OR "castigo físico*" OR "palmadas pedagógicas" OR "castigo corporal*" OR “打骂” OR “打屁股” OR “体罚” OR “العقاب البدني” OR “العقوبة البدنية” OR “العقاب الجسدي” OR “التأديب الجسدي” OR “التأديب بالعنف” OR “العقوبة بالعنف”
